# Health Literacy for Cardiac Rehabilitation: An Examination of Associated Illness Perceptions, Self-Efficacy, Motivation and Physical Activity

**DOI:** 10.3390/ijerph17228641

**Published:** 2020-11-20

**Authors:** Ronie Walters, Stephen J. Leslie, Jane Sixsmith, Trish Gorely

**Affiliations:** 1 Department of Nursing and Midwifery, Centre for Health Science, University of the Highlands and Islands, Inverness IV2 3JH, UK; stephen.leslie@nhs.scot (S.J.L.); trish.gorely@uhi.ac.uk (T.G.); 2 Cardiac Department, Raigmore Hospital, Inverness IV2 3UJ, UK; 3 Health Promotion Research Centre, National University of Ireland Galway, H91 TK33 Galway, Ireland; jane.sixsmith@nuigalway.ie

**Keywords:** health literacy, cardiac rehabilitation, rehabilitation participation, health behaviour, self-assessed health, remote and rural

## Abstract

Following a diagnosis of cardiovascular disease there is a need for patients to self-manage. Health literacy has been shown to be lower in patients with cardiovascular disease, yet research into health literacy in this population is limited. This study used the Health Literacy Questionnaire (HLQ) to examine the health literacy and associated health, health behaviours and psychological profiles of cardiac rehabilitation patients from a remote and rural regional programme in the Scottish Highlands. Consecutive patients referred to the service in a calendar year were sent a cross-sectional questionnaire by post. Hierarchical cluster analysis grouped respondents based on their health literacy profile, and nonparametric methods were used to analyse differences between clusters on the other measures. A total of 282 participants responded (45.7%). Respondents were older (median: 71 years) and more likely to be from more affluent areas. Five health literacy clusters emerged with different profiles of health, physical activity, self-efficacy, motivation and illness perceptions. There was no difference in relation to cardiac rehabilitation attendance by health literacy cluster, but those with lower health literacy were less likely to be aware of the referral. Patterns of health literacy are associated with health, health behaviours and some psychological constructs. Knowledge of distinct cluster characteristics may help services better target interventions.

## 1. Introduction

Cardiovascular diseases (CVDs), along with other noncommunicable diseases, are one of the leading causes of mortality [1] and morbidity worldwide [2]. Secondary prevention such as cardiac rehabilitation plays a vital role in reducing risk factors such as smoking, management of biophysical markers (e.g., cholesterol, blood pressure) and physical inactivity, which can be improved with interventions even after a cardiac event/CVD diagnosis. Cardiac rehabilitation has been shown to confer significant benefits including reduced mortality and improved quality of life [3,4,5,6]. However, the efficacy of cardiac rehabilitation depends on a number of factors, including effective referral pathways, accessible services and effective provision of information and support [7,8] that would enable people to access, understand and apply their learning in real world contexts.

In Scotland, 81.4% of CVD deaths have been attributed to modifiable risk factors that are influenced by health and wellbeing behaviours [9]. The UK was eleventh worst in Europe with regards to CVD incidence (age/sex adjusted per 100,000 of population), and amongst the four UK nations Scotland has the highest incidence of CVD [9]. The NHS Highland region, in the north of Scotland, covers 32,500 square kilometres, which makes it one of the largest health boards in the UK, serving a sparsely populated, predominantly remote and rural ageing population [10]. Geographical dispersion and poor infrastructure (e.g., roads and broadband) provide unique challenges compared with more urban settings. Statistics for cardiac rehabilitation uptake are not available for Scotland. Within the UK, data for England, Wales and Northern Ireland show uptake is consistently around 50% [11], and approximately a third of those do not complete cardiac rehabilitation [11]. Various reasons exist for this, including demographic (age, gender, education, work status), practical (geographical location, transport) and systemic (difficulty with access, poor referral pathways and low perceived need) factors [7,8]. Although many of these issues are beyond the reach of a cardiac rehabilitation programme to influence, health literacy has been shown to be modifiable at both individual and system level [12,13]. Taking an integrated approach [14] that incorporates action at both levels to develop a more health literacy-responsive service could potentially influence uptake, adherence and health outcomes.

Health literacy was described by Nutbeam as a multidimensional process composed of functional, interactive and critical health literacy [15]. As a complex social construct there are multiple definitions of health literacy, but many of them have been shown to include core dimensions relating to the ability to access, understand, evaluate and apply health information [16]. Whilst health literacy challenges can affect anyone, it follows a social gradient and is associated with the social determinants of health such as age, socio-economic status, education and being from an ethnic or linguistic minority [17,18,19,20]. It has also been shown to be linked to health indicators such as self-assessed health, health behaviour and long-term conditions [12,21]. Although figures differ by measure and construct, the prevalence of inadequate health literacy was estimated to be approximately 47% in Europe [17]. People with long-term conditions and/or multimorbidity have more health literacy challenges than the general population [20,22]. Amongst these, those with CVD have the most challenges with regards to understanding information and engaging with healthcare providers [20]. As a modifiable determinant, health literacy represents a key target for interventions on inequalities [12,13,23]. 

Whilst much early work focused on a narrow, functional approach—being concerned with people’s ability to read and write health information—it is becoming apparent that the focus on functional health literacy misses much of the richness and variability in people’s experiences. Measuring only a narrow aspect of capability such as literacy or numeracy misses other elements that could be protective factors for health. Health literacy is not a static construct with people having a single outcome—be that poor or good. People may have limitations in one aspect of health literacy (e.g., reading and understanding health information) but have compensatory strengths in other aspects (e.g., social support). The complex dynamic multidimensional nature of health literacy reflects the interaction between an individual’s competencies and the situational resources they have. The complexity and demands placed on an individual by organisations and systems are key to how well an individual can manage health literacy demands. 

Self-management after CVD is complex with patients being increasingly required to accomplish multiple different tasks [23]. Cardiac rehabilitation is known to help patients negotiate the self-management journey [24]. However, research into the health literacy of cardiac rehabilitation participants and the role of health literacy in attendance and health behaviours is limited. The majority of studies [25,26,27] have used single-scale measures that are unable to capture the full spectrum of participants’ relative strengths and weaknesses. This appears to be changing with two papers [28,29] published in 2020 using the Health Literacy Questionnaire (HLQ) [30] with cardiac rehabilitation patients in Australia and Denmark. These studies showed that health literacy was not associated with cardiac rehabilitation participation [29], but of those that did participate, people with higher levels of health literacy were more likely to drop out [28]. 

There is a need for services to understand the health literacy barriers their patients face to target interventions to support health literacy. Service provision and accessibility vary as a result of different health systems, cultures and contexts. Both Aaby et al. [29] and Beauchamp et al. [28] report on predominantly urban programmes taking place in a single centre. In contrast, this study focuses on a multicentre regional programme that covers a much greater geographical area. A previous study within this population [31] found people attending cardiac rehabilitation were more affluent, from less rural areas and had fewer morbidities. Multivariate analysis showed perceived need was a significant factor. Perceived need includes healthcare system factors (length of referral process, knowledge of cardiac rehabilitation, belief that healthcare providers support cardiac rehabilitation) and individual factors (perception of need, ability to self-manage, views on social norms). Many of these aspects could be influenced by unmet health literacy needs and therefore be a potential target for intervention design. 

As a multidimensional measure comprising nine individual subscales, the HLQ is well suited to examine health literacy within cardiac rehabilitation. It collects information on individual strengths and weaknesses, as well as perceived ideas of external factors such as healthcare provider support, social support and organisational aspects. Evaluating both internal and external factors supports the development of situationally relevant interventions. This can help organisations and clinicians to recognise and respond to individual difference in health literacy. For any intervention to be effective it needs to be targeted to the specific population and context [32]. A first step, therefore, is to undertake a mapping exercise of the population of interest. 

This study had two aims: (1) to profile the health literacy of cardiac rehabilitation patients using a cluster analysis of the HLQ and (2) to determine whether these clusters differ by health status, health behaviours, psychological profile, awareness of referral and participation in cardiac rehabilitation. This study will inform the development of an intervention framework to address the unmet health literacy needs of the patients of this cardiac rehabilitation service. 

## 2. Methods

### 2.1. Design and Data Collection

We screened the hospital medical records of 778 patients (an entire year of cardiac rehabilitation referrals from NHS Highlands) and identified 617 suitable for inclusion. Exclusion criteria included duplicate referrals within the year (n = 37), out-of-area patients (n = 58), those who were unsuitable to participate (too ill, unsuitable for cardiac rehabilitation, severe cognitive impairment (n = 30)) or those deceased (n = 36). We issued all 617 patients with a self-administered postal questionnaire and prepaid return envelope, with a follow-up issued to non-responders 3–4 weeks later. 

### 2.2. Measures

This study used previously validated measures with acceptable psychometric properties to collect data on health literacy, functional limitations, physical activity, illness perceptions, self-efficacy and motivation. Single-item, self-report questions collected information on demographics, cardiac rehabilitation attendance, health, smoking and alcohol consumption.

### 2.3. Health Literacy Measure

Health literacy was measured using the HLQ [30]. The questionnaire contains 44 questions in nine subscales, which can be used as standalone scales or as part of an overall package as in this study. The nine domains are as follows:

1. Feeling understood and supported by healthcare providers (HPS)
2. Having sufficient information to manage my health (HSI)
3. Actively managing my health (AMH)
4. Social support for health (SS)
5. Appraisal of health information (CA)
6. Ability to actively engage with healthcare providers (AE)
7. Navigating the healthcare system (NHS)
8. Ability to find good health information (FHI)
9. Understand health information well enough to know what to do (UHI)

Response options were on a four-point (domains 1–5) or five-point (domains 6–9) scale where higher scores represent higher health literacy. The expectation maximization algorithm was used to impute missing scores when no more than two items (scales with 4–5 items) or three items (scales with 6 items) were missing. Mean and standard deviations are reported for each scale. In addition, to facilitate comparison between all nine scales we calculated a percentage maximum score ((score/maximum possible) * 100).

### 2.4. Socio-Demographic Measures

Data collection included various socio-demographic data from medical records for both respondents and nonrespondents. This included gender, age and Scottish Index of Multiple Deprivation (SIMD) [33] derived from postcode. In addition, respondents indicated their highest level of education (school, college, undergraduate, postgraduate). For analysis, education was categorised as low education for school with all other further and higher education categories grouped together.

### 2.5. Health and Health Behaviour Measures

The diagnosis of both respondents and non-respondents was extracted from medical records. In addition, we extracted the presence of morbidities and we also asked respondents to indicate morbidities. Because the medical records only provided the most relevant morbidities on discharge from hospital, we used a combined metric and recorded a morbidity as present if it was reported by either the patient or the medical records.

Respondents also indicated their self-assessed health on a five-point scale from poor (1) through fair (2), good (3), very good (4) to excellent (5). For analysis, scores of one and two were grouped into a poor self-assessed health group, whilst scores of three and above were grouped into a good self-assessed health group. In addition, respondents indicated functional limitations using a validated six-item scale by Mora [34]. This scale asked participants to indicate how much difficulty they had with vigorous activities, moderate activities, walking, eating, things they would like to do and things they need to do. Items were scored on a five-point scale ranging from 1 (not at all) through to 5 (very much), where higher scores indicated greater functional limitations.

Additional information collected included body mass index (BMI, computed from self-reported height and weight), smoking status and frequency, alcohol consumption and physical activity. Physical activity was measured using the validated Stanford L-Cat [35] measure as it has been shown to be valid in mixed health literacy populations [36]. The L-Cat asks participants to indicate on a scale of 1–6 their usual physical activity over the past month. For analysis, responses of one to three were classed as not meeting UK physical activity guidelines, whilst responses of four or above were classed as likely meeting UK physical activity guidelines.

### 2.6. Psychological Measures

Illness perceptions were measured using the Revised Illness Perceptions Questionnaire (IPQ-R) [37]. For the purposes of this study we used the 7 subscales which measure timeline (acute/chronic), timeline (cyclical), personal control, treatment control, consequences, illness coherence and emotional representations. High scores on personal control, treatment control and illness coherence indicate positive beliefs regarding ability to influence the condition, the effectiveness of treatment and personal understanding of the condition. Conversely, high scores on timeline, consequences and emotional representations indicate beliefs regarding the chronicity and cyclical nature of the condition, as well as the negative consequences, and indicate a higher level of emotional distress when thinking about the condition.

To examine self-efficacy and motivation we focused on physical activity (as this health behaviour is a key focus in cardiac rehabilitation [38]). We included three self-efficacy scales [39] that measured confidence in ability to overcome barriers to participation in physical activity, confidence to do physical activity and confidence to schedule physical activity. All scales had between 3 and 4 items per scale and were scored from 1 (not at all) to 10 (completely). In addition, the Behavioural Regulations in Exercise Questionnaire (BREQ-3) [40] was included as a measure of motivation for physical activity. This measure contains 24 items scored from 1 (not true for me) to 5 (very true for me) and measures amotivation, external, identified, introjected, integrated, and intrinsic motivation.

### 2.7. Statistical Analysis

Hierarchical cluster analysis was conducted on all nine HLQ dimensions using Ward’s linkage on the Z-scores as the clustering method. Cluster analysis is a statistical approach recommended for use with the HLQ. It separates data into mutually exclusive groups composed of respondents with similar patterns of responses across the nine scales. The final number of clusters is determined by considering cluster size and response pattern variance. Reliability for individual subscales of the HLQ was acceptable with Cronbach’s alpha ranging from 0.77 (actively managing health) to 0.90 (navigating the healthcare system). For the other measures included, we calculated mean scores per measure and then used Kruskal–Wallis or chi-square (categorical data) to determine which measures were significantly different per cluster. Significance was set at *p* < 0.05. All measures had adequate reliability (Cronbach’s alpha > 0.71). We report means and standard deviations for normally distributed data, medians and interquartile range for non-normal distributions and percentages for categorical data. All statistical analyses were completed in SPSS version 25 [41].

### 2.8. Ethics and Approvals

All participants provided informed consent prior to completion of the questionnaire. The study was conducted in accordance with the Declaration of Helsinki, and ethical approval was granted on 23 March, 2019 by the Yorkshire and Humber-Leeds East REC committee (approval number 260464).

## 3. Results

In total, 282 patients completed the questionnaire out of 617 who were eligible (45.7% response rate). When compared with non-respondents, the respondents were similar in gender (male, 74.5% in responders vs. 68.1% in non-responders, *p* = 0.081), time since referral to rehabilitation (median of 9 (±6) months in both responders and non-responders, *p* = 0.898) and diagnosis (*p* = 0.435—see Table 1). Responders were older (median age 71 (±14) years in responders vs. 66 (±17) years in non-responders, *p* < 0.005) and more likely to be from more affluent areas (*p* < 0.005) (see Table 1). Six participants had insufficient data on the HLQ to allow sorting into clusters and so were excluded from further analysis.

Mean scores for all HLQ scales are shown in Table 2. For scales 1 to 5, the highest score was for scale 4, social support (3.02 ± 0.50), and the lowest was scale 5, critical appraisal (2.57 ± 0.54). For scales 6–9, the highest score was for scale 9, reading and understanding health information (3.98 ± 0.62), and the lowest was scale 7, navigating the healthcare system (3.73 ± 0.68).

Respondents were grouped into clusters based on the health literacy scores. Consideration of response patterns, cluster size and standard deviation suggested a five-cluster solution as the most appropriate. The cluster with the lowest scores was a very small cluster (18 respondents), which persisted even if we reduced the number to three or increased to eight clusters, so we determined it was an important sector of the population and therefore included it. 

Consistent with guidance for using the HLQ with cluster analysis to profile subgroups within populations [42], we used excel conditional formatting to colour code across clusters within scales 1–5 and scales 6–9. Conditional formatting shades the highest score in the range green, the lowest red and the median yellow. All other cells are shaded proportionally to provide a visual guide to relative strengths and weaknesses. This allows us to see variations both within clusters and across health literacy domains. For example, we can see that cluster 1 is generally stronger across all domains and cluster 5 is generally weaker. Within scales we can see that critical appraisal is generally the weakest domain, though in this instance cluster 4 is stronger than cluster 3.

To facilitate easier comparison between scales using different scoring, we converted the scores to percentage maximum, as shown in Table 3. We also reordered them from left to right to show strongest (scale 9) to weakest (scale 5) based on overall sample mean. The use of conditional formatting applied within each individual cluster allows easier comparison of patterns of relative strengths and weaknesses within clusters compared with the overall mean. For example, scale 3 (actively managing health) is a relative strength for clusters 4 and 5 but it is the second weakest for the overall sample mean.

Table 4 shows the scores of the overall sample, as well as the five clusters across the demographic, health, health behaviour and psychological scales used in the study. Of the respondents, 52% had completed only secondary school level education. With regards to health and health behaviours, 23.4% reported poor or fair health, with a median functional limitation score of 2.17 (out of 5), and the median number of morbidities (including cardiac) was 4.00. The median BMI was 26.95, with 25.2% being obese. In terms of alcohol consumption, 70.1% still drank alcohol and 21.3% exceeded the weekly maximum recommended 14 units. Almost half (48.6%) had given up smoking, and just 8.1% still smoked. Finally, 44% were unlikely to be meeting the UK physical activity guideline of 150 min of moderate (or 75 min vigorous) physical activity per week (see Table 2). The sample had strongest self-efficacy for doing physical activity (median 8.50), were more likely to have identified motivation (median 4.25) and reasonably strong illness coherence (median 4.00) and acute/chronic (median 3.83) beliefs. 

Comparison of the subgroups (clusters 1–5) showed no significant difference in terms of age, gender, education or Scottish Index of Multiple Deprivation. Nor did they differ in relation to smoking or alcohol consumption (see Table 4), so these will not be described further. Using Table 2, Table 3, Table 4, we can see that cluster 1, n = 54 (20% of the sample), could be described as “motivated and supported”. People in Cluster 1 have the best health out of this sample population with 92.6% reporting good–excellent health, and just 4.2% of them reporting considerable functional limitations. Only 11.1% were unaware of the cardiac rehabilitation referral. They have good social support (scale 4) and can engage effectively with healthcare providers (scale 6) to ensure they get what they need and make sure their questions are answered. They feel like the condition makes sense to them (illness coherence) and feel that the treatment is effective (treatment control) and that what they do will make a difference (personal control). With the highest levels of intrinsic motivation and self-efficacy, they are physically active with almost 75% of them meeting or exceeding the guidelines.

Cluster 2, n = 76 (28% of the sample), could be described as “go it alone triers”. With slightly fewer morbidities per person, less people experiencing functional limitations (3.2%) and 87.8% of people having good–excellent health, they are very similar to Cluster 1, though slightly more of them are obese (18.7%). They have less effective support from others (scales 1 and 4) although they are good at understanding health information (scale 9) and engaging effectively to ensure they get the information they need from healthcare providers (scale 6). They do not believe their illness will keep returning (cyclical), nor do they think it causes negative consequences or causes them sadness, anger or fear (emotional representations). Whilst 61% of them do meet the UK physical activity guidelines, they have lower levels of motivation and self-efficacy than Cluster 1.

Cluster 3, n = 58 (21% of the sample), could be described as “accepting”. The middle cluster has significantly worse health than the two highest clusters with 61.4% reporting good–excellent health and the highest BMI of the study (29.16) with 36.8% being obese. Like Cluster 2, they are good at understanding health information (scale 9) and engaging with healthcare providers (scale 6), but they struggle to apply information to actively manage their own health (scale 3). They have lower motivation and self-efficacy (particularly with regards to organising physical activity) and they believe their condition to be cyclical and so likely to return. However, this does not particularly upset or anger them (emotional representations).

Cluster 4, n = 66 (24% of the sample), could be described as “active worriers”. With just 2% difference in overall health literacy compared with Cluster 3, those in Cluster 4 are generally healthier and more active with a lower BMI. They are good at understanding health information (scale 9), but unlike cluster 3 they are better at applying the information to manage their health (scale 3). They are not as good at engaging with healthcare providers (scale 6) to ensure they have all the information they need, and they are also weaker at being able to find the help they need (scale 7) with 30.3% being unaware of the referral to cardiac rehabilitation. They are less likely to understand their condition (illness coherence), considering it to be cyclical with negative consequences (cyclical and consequences). They also are less likely to believe anything they do will make a difference (personal control) and so experience negative emotions when thinking about their condition (emotional representations).

Cluster 5, n = 18 (7% of the sample), could be described as “poorly and challenged”. The final cluster has poor health (61.1% report poor or fair health) with the highest number of morbidities and almost a third of them experiencing considerable functional limitations. Even though their health literacy is low across the board, it appears they are able to apply information to manage their health (scale 3) with the support of friends and family (scale 4), but are likely to struggle to find information, find who they need to see or know what help they are entitled to (scales 7 and 8). This may mean they are less engaged with healthcare services (scale 6). Half (50%) of the people in this cluster were unaware of the referral to cardiac rehabilitation. They are the least active cluster with 68.8% of them unlikely to be meeting UK physical activity guidelines. They are amotivated with low self-efficacy and low intrinsic motivation. They think their condition is likely to return (cyclical) and they do not really understand it (illness coherence). They get upset or afraid when they think about it (emotional representations) and they do not think treatment will help (treatment control) or that they can influence what happens (personal control).

## 4. Discussion

This study examined the health literacy profiles of a single-year cohort of patients referred to cardiac rehabilitation in the remote and rural Highlands region of Scotland. Five distinct profiles of health literacy emerged, which were associated with gradually decreasing levels of self-assessed health and increasing number of morbidities and functional limitations. The clusters did not differ with respect to SIMD, gender, age, education, alcohol consumption, smoking or cardiac rehabilitation attendance. There were however distinct differences in relation to awareness of the cardiac rehabilitation invite, with 30–50% of those in Clusters 4 and 5 failing to recognise they had been invited. In addition, the profiles differed on BMI (with Cluster 3 having significantly higher BMI) and physical activity. Clusters with more restricted health literacy were less physically active, less motivated, with lower self-efficacy for physical activity and more negative and distressing illness perceptions.

The impact of health literacy on cardiac rehabilitation attendance is complex and still not fully understood. This could be because the ability to engage with cardiac rehabilitation is complex and influenced by broader societal influences that are often beyond the individual’s control. Our study supports recent findings by Aaby [29] that showed that neither socio-demographic factors nor health literacy were associated with cardiac rehabilitation participation. However, we did find that clusters differed with respect to awareness of the referral. Our sample was an entire year of patients that the cardiac rehabilitation team had received a referral for and contacted. Patients in Clusters 4 and 5 were less likely to recognise they had been referred than those in Cluster 1. Initial access and subsequent navigation of the healthcare system was a relative weakness for Clusters 4 and 5, and this indicates that interventions to target the referral process could be effective at increasing uptake. 

This study found a more mixed picture with regards to the association between health literacy clusters and demographic, health and behavioural outcomes. In line with other studies [20], we found clear associations between health literacy cluster and self-assessed health, morbidities and functional limitations. In contrast to previous studies reporting an association with education [12,20], we found no statistically significant differences between the clusters on education (*p* = 0.172). We did however see a trend for those with lower health literacy to be more likely to have completed only secondary school level education. This could be due to the lack of sensitivity within the measure as the question was unable to differentiate between those who left school early and those who achieved standard or higher-level qualifications. We were also unable to confirm a link between health literacy and socio-economic status (*p* = 0.501) in this study, however the use of a composite area-based measure (SIMD) that includes factors such as income, crime, rurality and education may have masked associations with individual-level socio-economic status. With regards to health behaviours, we were able to confirm findings by Svendsen et al. [21], who found no association between health literacy and smoking and alcohol, but that health literacy was associated with physical activity. We are able to extend this finding by adding psychological measures that show distinct differences in self-efficacy, motivation and illness perceptions between clusters when patients are grouped solely on health literacy responses. This shows promise as an effective avenue for self-management interventions, as acceptance of the condition has previously been shown to mediate health literacy in self-management of hypertension [43]. 

### 4.1. Implications

As a person-centred process, there is a clear need for cardiac rehabilitation to consider health literacy barriers and profiles to better target the provision of services to meet patient’s needs [44,45,46]. The HLQ benefits this process by identifying distinct clusters with different patterns of health literacy strengths and weaknesses. This approach allows us to take a whole-sample view, but also identify variation within the sample. For example, although active engagement is a strength in the overall group, it is a relative weakness for Clusters 4 and 5. We can also begin to consider possible interactions between skills and how they might help or hinder patients. Being able to identify what stronger clusters are doing well compared with the more restricted clusters could allow us to better target interventions.

Although evidence for the benefit of health literacy interventions in cardiac rehabilitation is limited, a recent systematic review [47] found two successful interventions with cardiac patients, and additionally that in seven out of eight studies (any population) health literacy interventions had a positive effect on health behaviours. However, it is worth considering that many health literacy interventions still focus on functional health literacy and making information easier to understand. Recent work with the HLQ suggests that (at least in Scotland and Australia) understanding information is not the biggest challenge. Information processing was a relative strength for most of our clusters. Challenges were most commonly seen with the more action-oriented aspects such as critical appraisal, accessing and navigating the healthcare system and actively managing health. This mirrors findings in health literacy and self-management research that indicate a need for interventions to focus on action aspects and supportive relationships [45,46,48]. 

Whilst this study has shown distinct patterns of health literacy within the cardiac population in the Highlands region, identifying people from specific clusters is not easy to do in practice. None of the demographic elements were predictive of health literacy cluster membership. Although screening can be undertaken, there is not yet a concise method that can capture the complexity of health literacy within the time constraints of clinical appointments [49] and analysis of the HLQ is too complex to allow easy categorisation within clinical practice. In addition, the use of screening can cause embarrassment and shame for the patient [50] and risks leading to a focus on health literacy as a deficit of the patient rather than a collaborative asset to be built between patients and healthcare services. Given the links between health literacy, healthcare provider support and active engagement, there is a clear need for healthcare providers to be confident in health literacy approaches. Brooks [51] notes that even though many healthcare providers recognise the importance of addressing health literacy, they do not always have confidence in commonly recommended strategies. They are also wary of the universal precautions approach for risk of oversimplifying things for those with stronger health literacy. Training healthcare providers in health literacy has been shown to be effective at improving knowledge, attitudes and skills relating to health literacy [52]. Given the need to target approaches to support the different profiles of need in the clusters, future steps to support implementation in practice will benefit from the inclusion of staff in line with the OPHELIA (OPtimising HEalth LIterAcy) approach [53].

### 4.2. Strengths and Limitations

This study has added to the body of knowledge around health literacy in cardiac rehabilitation by using the HLQ and cluster analysis to build a multidimensional picture of health literacy strengths and weaknesses. We have further extended this contribution by adding psychological and behavioural measures to better reflect the complexity of cardiac rehabilitation and show how they interact with different health literacy profiles. 

The use of self-report, cross-sectional, mailed questionnaires may limit the generalisability of our findings. Self-report measures can be skewed depending on the accuracy of participant perceptions, but research by Hawkins et al. [54] showed reasonable congruence between patients reasoning and HLQ intentions, as well as between patient and provider. The addition of psychological measures provided us with valuable insight into participants but may have added to the burden to participants. Our response rate was respectable for a postal survey, however respondents differed in relation to age (being older) and SIMD (being from more affluent areas). Previous research suggests health literacy can be lower in older participants and higher in those from more affluent areas, so this may have contributed to the lack of association between health literacy and demographics in this study. Finally, this study only recruited from the NHS Highland service, which has quite unique geographical and infrastructure constraints. This may limit generalisability to other contexts both within Scotland/UK and in the wider global context. Nevertheless, the similar patterns observed in other recently published studies in cardiac rehabilitation patients in more urban settings suggest some commonalities of experience and lends strength to our findings. 

## 5. Conclusions

Our results show that when grouping patients by health literacy, differences also emerge in relation to self-assessed health, physical activity, self-efficacy, motivation and illness perceptions. The groups follow a gradient, with those with more restricted health literacy being less physically active and having poorer health, and more distressing illness perceptions. Our results did not show an association between health literacy and cardiac rehabilitation attendance, but those in the lowest two clusters were significantly less likely to be aware they had been referred to cardiac rehabilitation. This study extends recent work into health literacy in this population and can be used to inform future development of interventions.

## Figures and Tables

**Table 1:** Baseline population characteristics.

		Respondents(n = 282) 45.71%		Non-Respondents(n = 335)		*p*-Value
Age (Median ± IQR) range		(71 ± 14) 33–94		(66 ± 17) 28–93		***p* < 0.005**
Gender	Male	210	(74.5%)	228	(68.1%)	*p* = 0.081
	Female	72	(25.5%)	107	(31.9%)	
Scottish IndexMultiple Deprivation	1 (most deprived)	8	(2.8%)	20	(6.0%)	***p* < 0.005**
	2	38	(13.5%)	86	(25.7%)	
	3	92	(32.6%)	102	(30.4%)	
	4	91	(32.3%)	97	(29.0%)	
	5 (least deprived)	53	(18.8%)	30	(9.0%)	
Months since referral (Median ± IQR) range		(9.00 ± 6.00) 2–15		(9.00 ± 6.00) 2–15		*p* = 0.898
Diagnosis	NSTEMI *	115 (34.3)		89 (31.6)		*p* = 0.435
	STEMI **	80 (23.9)		58 (20.6)		
	Unstable angina	22 (6.6)		26 (9.2)		
	Stable angina	70 (20.9)		69 (24.5)		
	Heart failure	6 (1.8)		9 (3.2)		
	Structural cardiac disease	28 (8.4)		24 (8.5)		
	Other	14 (4.2)		7 (2.5)		

* NSTEMI = non-segment elevated myocardial infarction; ** STEMI = segment elevated myocardial infarction. **Bold** denotes significant result.

**Table 2:** Cluster analysis.

Cluster	Count of ID	% in cluster	Scale 1	Scale 2	Scale 3	Scale 4	Scale 5	Scale 6	Scale 7	Scale 8	Scale 9
			HPS	HSI	AMH	SS	CA	AE	NHS	FHI	UHI
**All: Mean**			3.00	2.96	2.92	3.02	2.57	3.88	3.75	3.73	3.98
1	54	20	3.66	3.52	3.31	3.66	3.02	4.58	4.35	4.31	4.56
2	76	28	3.02	3.08	2.95	3.02	2.73	4.23	4.13	4.12	4.23
3	58	21	2.98	2.89	2.61	2.93	2.13	3.92	3.61	3.53	3.90
4	66	24	2.68	2.66	2.97	2.80	2.59	3.34	3.27	3.40	3.67
5	18	7	2.21	2.18	2.51	2.27	1.96	2.36	2.30	2.32	2.67

HPS = Healthcare Provider Support, HIS = Having Sufficient Information, AMH = Actively Managing Health; SS = Social Support; CA = Critical Appraisal; AE = Active Engagement; NHS = Navigating Healthcare System; FHI = Finding Health Information; UHI = Understanding Health Information. [Colour coding represents strength on scale with green = higher scores and red = lower scores.]

**Table 3:** Maximum percentage scores.

	Scale 9	Scale 6	Scale 4	Scale 1	Scale 7	Scale 8	Scale 2	Scale 3	Scale 5
	UHI	AE	SS	HPS	NHS	FHI	HSI	AMH	CA
All	79.60	77.60	75.50	75.00	75.00	74.60	74.00	73.00	64.25
1	91.20	91.60	91.50	91.50	87.00	86.20	88.00	82.75	75.50
2	84.60	84.60	75.50	75.50	82.60	82.40	77.00	73.75	68.25
3	78.00	78.40	73.25	74.50	77.20	70.60	72.25	65.25	53.25
4	73.40	66.80	70.00	67.00	65.40	68.00	66.50	74.25	64.75
5	53.40	47.20	56.75	55.25	46.00	46.40	54.50	62.75	49.00

HPS = Healthcare Provider Support, HIS = Having Sufficient Information, AMH = Actively Managing Health; SS = Social Support; CA = Critical Appraisal; AE = Active Engagement; NHS = Navigating Healthcare System; FHI = Finding Health Information; UHI = Understanding Health Information. [Colour coding represents strength on scale with green = higher scores and red = lower scores.]

**Table 4:** Sample and cluster medians for health, behaviour and psychological measures.

	Overall	Cluster 1	Cluster 2	Cluster 3	Cluster 4	Cluster 5	Significance
	Median (IQR)	Median (IQR)	Median (IQR)	Median (IQR)	Median (IQR)	Median (IQR)	
Demographics							
Age	71 (14)	69 (10)	70 (14)	73 (10)	70 (13)	71 (17)	*p* = 0.540
Male gender	74.50%	66.70%	81.60%	75.90%	72.70%	61.10%	*p* = 0.242
Scottish Index Multiple Deprivation	3 (1)	3 (1)	3 (1)	3 (1)	3 (1)	3 (2)	*p* = 0.501
School only education	51.80%	46.30%	47.40%	55.20%	51.50%	77.80%	*p* = 0.172
Unaware of invite	20.40%	**11.1%^↓^**	18.90%	12.10%	**30.3%^↑^**	**50%^↑^**	***p* = 0.001**
Did not attend	46%	40.40%	40.30%	38.90%	55.40%	66.70%	*p* = 0.129
Health							
Morbidities	4.00 (2.00)	4.00 (2.00)	**4.00^↓^ (2.00)**	4.00 (3.00)	4.00 (3.00)	**5.00^↑^ (3.25)**	***p* = 0.020**
Percentage with poor/fair self-assessed health	23.40%	**7.4%^↓^**	**12.2%^↓^**	**38.6%^↑^**	25.80%	**61.1%^↑^**	***p* < 0.0005**
Functional limitations	2.17 (1.67)	**1.67^↓^ (1.17)**	**1.50^↓^ (1.17)**	2.83 (1.63)	2.33 (1.46)	**3.08^↑^ (1.38)**	***p* < 0.0005**
Percentage with functional limitation score 4 /5	9.80%	4.20%	3.20%	15.40%	10%	31.30%	
Body Mass Index (BMI)	26.95 (5.23)	**26.82^↓^ (3.79)**	**26.10^↓^ (3.95)**	**29.03^↑^ (6.12)**	26.84 (6.28)	26.80 (9.44)	***p* =0.012**
Percentage with BMI ≥30	25.20%	17.30%	18.70%	36.80%	27.70%	29.40%	
Health Behaviours							
Alcohol units	5.00 (10.52)	5.20 (13.20)	5.63 (13.45)	4.5 (7.98)	4.75 (10.00)	9.00 (37.00)	*p* = 0.536
Percentage drinking >14 units per week	21.30%	26.80%	26.90%	13.60%	15.90%	28.60%	
Percentage smoking	8.10%	5.60%	9.40%	12.10%	3.10%	16.70%	*p* = 0.055
Percentage not meeting UK physical activity guidelines	44%	**26.4%^↓^**	**38.9%^↓^**	**58.9%^↑^**	45%	**68.8%^↑^**	***p* < 0.005**
Self-efficacy							
Confidence in ability to overcome	6.60 (3.20)	**7.80^↑^ (2.40)**	6.80 (3.15)	**5.70^↓^ (4.70)**	6.60 (2.60)	**4.20^↓^ (5.30)**	***p* < 0.0005**
Confidence Physical Activity	8.50 (3.25)	**9.63^↑^ (1.56)**	9.00 (2.44)	8.13 (3.69)	7.75 (2.38)	**5.75^↓^ (5.50)**	***p* < 0.0005**
Confidence scheduling	7.00 (4.08)	**8.50^↑^ (2.42)**	**7.67^↑^ (3.17)**	6.33 (4.17)	7.00 (3.25)	**2.50^↓^ (3.75)**	***p* < 0.0005**
Motivation							
Amotivation	1.00 (0.00)	1.00 (0.00)	1.00 (0.00)	1.00 (0.50)	1.00 (0.00)	**1.50^↑^ (1.38)**	***p* < 0.0005**
Extrinsic	1.66 (1.00)	1.5 (1.13)	1.13 (1.44)	1.5 (1.00)	1.25 (1.00)	1.25 (0.75)	*p* = 0.837
Introjected	3.00 (1.75)	**3.00^↑^ (1.38)**	3.00 (1.81)	**2.75^↓^ (1.69)**	3.00 (1.00)	**2.00^↓^ (1.88)**	*p* = 0.036
Identified	4.25 (1.25)	**4.50^↑^ (0.75)**	4.25 (1.44)	**4.00^↓^ (1.50)**	4.00 (1.00)	**3.00^↓^ (1.19)**	***p* < 0.0005**
Integrated	3.88 (2.25)	**4.50^↑^ (1.50)**	4.25 (2.50)	**2.75^↓^ (3.00)**	3.75 (1.50)	**2.13^↓^ (1.80)**	***p* < 0.0005**
Intrinsic	4.00 (2.00)	**4.50^↑^ (1.00)**	4.00 (1.69)	**3.50^↓^ (1.75)**	3.88 (1.50)	**3.00^↓^ (0.81)**	***p* < 0.0005**
Illness perceptions							
Timeline	3.83 (1.33)	3.83 (1.25)	3.67 (1.33)	4.00 (1.17)	3.92 (1.33)	4.00 (1.25)	*p* = 0.158
Cyclical	2.25 (1.00)	**2.00^↓^ (1.13)**	**2.00^↓^ (0.31)**	**2.25^↑^ (1.00)**	**2.50^↑^ (1.27)**	**2.88^↑^ (1.50)**	***p* < 0.0005**
Consequences	3.00 (1.20)	2.60 (1.40)	**2.40^↓^ (1.20)**	3.00 (1.60)	**3.20^↑^(1.00)**	3.20 (1.05)	***p* < 0.0005**
Personal control	3.83 (0.83)	**4.17^↑^ (0.88)**	3.83 (0.50)	3.67 (0.67)	**3.55^↓^ (0.67)**	**3.25^↓^ (1.08)**	***p* < 0.0005**
Treatment control	3.60 (0.80)	**3.80^↑^ (0.85)**	3.60 (0.80)	3.60 (0.90)	3.50 (0.60)	**3.00^↓^ (0.90)**	***p* < 0.0005**
Illness coherence	4.00 (0.80)	**4.70^↑^ (1.00)**	4.00 (0.60)	4.00 (0.55)	**3.70^↓^ (0.80)**	**3.40^↓^ (1.45)**	***p* < 0.0005**
Emotional representation	2.33 (1.17)	**2.17^↓^ (1.54)**	**2.33^↓^ (0.83)**	**2.50^↓^ (1.04)**	**2.83^↑^ (1.25)**	**2.92^↑^ (1.67)**	***p* < 0.0005**

**Bold** = significant result; ^↓^ denotes a cluster significantly lower than other clusters in post hoc testing. ↑ denotes a cluster significantly higher than other clusters in post hoc testing.
